# GenAI literacy and perceived decision quality in e-commerce: the mediating role of perceived information overload and the moderating role of consumer involvement

**DOI:** 10.3389/fpsyg.2026.1919451

**Published:** 2026-10-21

**Authors:** Yiming Guo

**Affiliations:** School of Information, Central University of Finance and Economics, Beijing, China

**Keywords:** consumer involvement, GenAI literacy, generative artificial intelligence, perceived decision quality, perceived information overload

## Abstract

Generative artificial intelligence (GenAI) is increasingly used to support product search, comparison, and evaluation in e-commerce. However, consumers differ in their ability to use and critically evaluate GenAI-generated information, which may shape their perceived information overload and decision outcomes. Grounded in Information Processing Theory, this study examines the association between GenAI literacy and perceived decision quality, the mediating role of perceived information overload, and the moderating role of consumer involvement. Data were collected through an online survey of 401 Chinese college students and analyzed using confirmatory factor analysis and bootstrapped mediation and moderation procedures. The results show that GenAI literacy is positively associated with perceived decision quality and negatively associated with perceived information overload. Perceived information overload is negatively associated with perceived decision quality and accounts for a significant indirect association between GenAI literacy and perceived decision quality. Consumer involvement further moderates the association between GenAI literacy and perceived information overload: the negative association is stronger at higher levels of involvement. These findings support an information-processing account in which consumers’ GenAI-related capabilities and motivational involvement jointly shape their perceptions of cognitive burden and decision quality. The study highlights the importance of strengthening consumers’ critical GenAI literacy and adapting AI-supported information presentation to different levels of consumer involvement.

## 1 Introduction

Generative Artificial Intelligence (GenAI) has become part of the information environment in which consumers search for, compare, and evaluate products. In online shopping, conversational GenAI systems can respond to follow-up questions, summarize product attributes, and generate recommendations based on the wording and context of a user’s request. This form of interaction differs from conventional search and recommendation interfaces, where consumers typically select among links or options presented by the system. Research on consumer interactions with AI has shown that the design of these systems can influence consumer autonomy, trust, satisfaction, and product evaluation (Puntoni et al., 2021; Han and Ko, 2025; Kim and Priluck, 2025).

The capacity of GenAI to synthesize large amounts of product information may reduce the effort required to locate and organize relevant material. The resulting information still requires evaluation. Generated responses may be lengthy, vary across prompts, or provide recommendations without making their evidential basis clear. Fluent and coherent responses can also contain inaccurate or unsupported claims (Alkaissi and McFarlane, 2023). Consumers must consequently judge which information is relevant, how much confidence to place in it, and whether additional verification is warranted. These activities draw on limited attention and working-memory resources. Information Processing Theory (IPT) offers a useful basis for examining this problem because it explains how the amount, complexity, and ambiguity of information can exceed an individual’s processing capacity and produce a sense of overload (Arnold et al., 2023).

Consumers vary in their ability to manage these demands. GenAI literacy encompasses the knowledge and skills required to understand AI systems, use them effectively, evaluate their outputs, and recognize relevant ethical considerations (Long and Magerko, 2020; Ng et al., 2021). Consumers with stronger GenAI literacy may be better equipped to formulate precise requests, identify uncertain claims, and organize generated information around the requirements of a purchase decision. Emerging evidence nevertheless shows an uneven relationship between familiarity with AI and the quality of users’ evaluations. Lower AI literacy has been associated with greater receptivity to AI-generated recommendations (Tully et al., 2025), while AI-supported task performance can exceed users’ ability to assess their own performance accurately (Fernandes et al., 2026). These findings make it important to examine how GenAI literacy relates to the cognitive burden consumers experience and to their evaluation of the resulting decision.

Existing studies of AI and consumer behavior have examined trust, acceptance, autonomy, personalization, and responses to AI-based recommendations. Research on AI-integrated e-commerce has also identified cognitive and social factors as relevant to consumer and platform outcomes (Ahmed et al., 2024), while recent work on AI exposure in online shopping has linked consumers’ responses to trust, privacy concerns, emotional reactions, and decision processes (Ahmed et al., 2026). Less attention has been given to the relationship among GenAI literacy, perceived information overload, and perceived decision quality within a single explanatory model. Consumer involvement may further shape this relationship because the importance attached to a purchase affects the effort that consumers are willing to devote to processing and verifying information. Examining these variables together can clarify why consumers who use similar GenAI tools may evaluate their decision experiences differently.

Drawing on IPT, this study examines survey data from 401 Chinese university students to investigate the relationship between GenAI literacy and perceived decision quality in GenAI-assisted online shopping. The proposed model considers both the direct association between these constructs and the indirect association through perceived information overload. It also examines whether consumer involvement conditions the relationship between GenAI literacy and perceived information overload. By bringing individual capability, perceived processing burden, and task involvement into the same model, the study extends research on consumer adoption of AI toward the cognitive processes involved in evaluating AI-assisted purchase decisions. The following sections develop the theoretical framework and hypotheses, describe the research design, report the empirical results, and discuss the implications and limitations of the findings.

## 2 Literature review and hypothesis development

### 2.1 Literature review

#### 2.1.1 Consumer information processing in GenAI-assisted shopping

Consumer decision-making involves selecting relevant information, interpreting its meaning, and integrating different cues into an evaluation of available alternatives. Information Processing Theory (IPT) assumes that these activities take place under limited cognitive capacity. Attention and working memory constrain the amount and complexity of information that individuals can process at a given time (Sweller, 2011). In online shopping, extensive product assortments, repetitive descriptions, and conflicting reviews can place substantial demands on these resources. Information overload develops when consumers feel unable to process the available information adequately within the time and effort they can devote to the decision (Li, 2014; Arnold et al., 2023).

GenAI changes the organization and presentation of product information. Conversational systems can summarize product attributes, compare alternatives, answer follow-up questions, and generate recommendations in response to a consumer’s stated preferences. These functions can reduce the effort involved in locating and organizing information. At the same time, the consumer must evaluate a system-produced synthesis whose sources and selection criteria may not always be visible. Generated responses can also remain fluent and persuasive when they contain inaccurate or unsupported claims (Alkaissi and McFarlane, 2023). The cognitive demands of GenAI-assisted shopping therefore arise from both the information presented and the judgments required to establish its relevance and credibility.

Studies comparing GenAI chatbots with conventional search engines show that conversational interaction changes how consumers approach product evaluation (Kim and Priluck, 2025). The framing and breadth of generated recommendations can also influence consumers’ sense of empowerment and the desirability of consumption goals (Richarde et al., 2025). Related research on AI-integrated e-commerce has identified cognitive and social factors as part of the routines through which AI shapes online consumer activity (Ahmed et al., 2024). Research on AI exposure in online shopping similarly connects consumer responses with trust, privacy concerns, emotional reactions, and decision processes (Ahmed et al., 2026). This body of work places the consumer’s information-processing capacity at the center of understanding how GenAI is used during product choice.

#### 2.1.2 GenAI literacy as an information-processing capability

AI literacy describes the knowledge and skills required to understand AI systems, interact with them effectively, evaluate their outputs, and recognize the ethical implications of their use (Long and Magerko, 2020). Ng et al. (2021) organize these capabilities around understanding, application, evaluation, and ethics. More recent research has adapted this general concept to GenAI. The resulting frameworks include competencies related to information and data literacy, content generation, communication, problem-solving, safety, and ethical awareness (Gökçearslan et al., 2024; Lee et al., 2026). Although their dimensions vary, these frameworks consistently treat critical evaluation as part of competent GenAI use.

Within IPT, GenAI literacy can function as a set of knowledge structures and practical strategies that guide the allocation of limited cognitive resources. Consumers who understand the capabilities and limitations of GenAI can formulate more focused requests, identify relevant comparison criteria, and recognize outputs that require further checking. They may also be better able to separate useful product information from repetition, unsupported claims, or details unrelated to the purchase objective. These skills can make interactions with GenAI more organized and reduce the effort required to interpret generated responses.

The behavioral consequences of AI literacy are more complex than greater acceptance of AI advice. Tully et al. (2025) found that consumers with lower AI literacy displayed greater receptivity to AI-generated recommendations. Fernandes et al. (2026) showed that AI-assisted performance and metacognitive accuracy can diverge, with users benefiting from AI while remaining inaccurate in their assessment of their own performance. These findings indicate that receptivity, confidence, and evaluative competence represent different aspects of human–AI interaction. In consumer settings, GenAI literacy is therefore relevant to both the cognitive demands experienced during information processing and consumers’ assessments of the decisions reached with AI assistance.

#### 2.1.3 Perceived information overload and perceived decision quality

Perceived information overload arises when individuals feel that the demands of processing available information exceed their cognitive capacity (Arnold et al., 2023; Hunter et al., 2024). In conventional online shopping, this experience is commonly associated with large product assortments, repetitive descriptions, and conflicting reviews. GenAI-assisted shopping introduces additional sources of cognitive demand. Generated responses may combine information from multiple sources, present numerous comparison criteria, or offer recommendations whose factual basis is not immediately visible. Because fluent and coherent outputs can still contain inaccurate or unsupported claims, consumers may need to assess the relevance, consistency, and credibility of the information before incorporating it into a purchase decision (Alkaissi and McFarlane, 2023). This continuing verification effort adds to the cognitive work already involved in comparing alternatives and may heighten consumers’ perceptions of information overload.

Perceived decision quality captures consumers’ evaluations of the decision process and its outcome, including their confidence in the final choice and their perception that relevant alternatives have been considered systematically (Chae and Lee, 2013). From the perspective of IPT, such evaluations depend partly on whether consumers can allocate sufficient attention to identifying relevant attributes, comparing options, and resolving inconsistent information. When information-processing demands become excessive, consumers may narrow the range of alternatives considered, overlook potentially useful information, or rely more heavily on simplifying heuristics. These responses can weaken confidence in the decision and reduce the perceived adequacy of the choice process. The issue is especially relevant in GenAI-assisted settings because language models may reproduce familiar judgment biases, while users may remain highly confident in AI-supported decisions even when their assessment of actual performance is inaccurate (Chen et al., 2025; Fernandes et al., 2026).

The relationship between perceived information overload and perceived decision quality also provides a theoretical link between GenAI literacy and consumer decision outcomes. Consumers with stronger GenAI literacy are likely to possess more developed strategies for formulating queries, filtering generated information, identifying questionable claims, and determining when additional verification is necessary. These capabilities may help them maintain a more manageable information-processing environment and support a more systematic evaluation of purchase alternatives. Existing studies have examined AI literacy, information overload, and consumer responses to AI largely as separate issues, leaving their combined role in GenAI-assisted shopping insufficiently understood. Building on IPT, the present study brings these elements together by examining the association between GenAI literacy and perceived decision quality, the mediating role of perceived information overload, and the extent to which consumer involvement conditions this process.

### 2.2 Hypothesis development

The conceptual model distinguishes four elements of GenAI-assisted consumer decision-making. GenAI literacy represents an individual capability relevant to the use and evaluation of generated information. Perceived information overload captures the cognitive demands experienced during that process, while perceived decision quality reflects consumers’ assessment of the resulting choice. Consumer involvement represents the motivational importance attached to the purchase decision. The following hypotheses specify the expected relationships among these constructs. The proposed conceptual model is presented in Figure 1.

**Figure 1:**
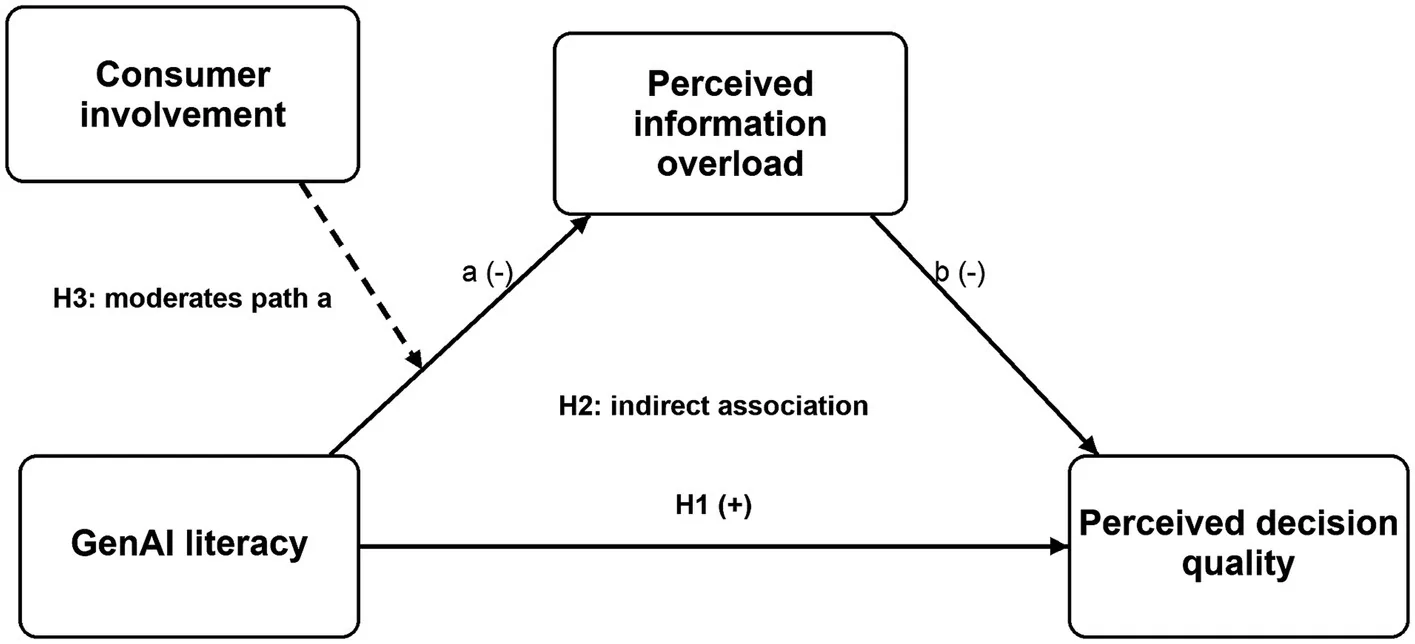
Conceptual model of the proposed relationships among GenAI literacy, perceived information overload, perceived decision quality, and consumer involvement. Consumer involvement moderates path a, the first stage of the proposed indirect association.

#### 2.2.1 GenAI literacy and perceived decision quality

IPT suggests that prior knowledge and task-relevant skills help individuals identify useful information and organize it into a form that can be processed efficiently. In GenAI-assisted shopping, literacy provides consumers with a basis for formulating appropriate requests, interpreting generated responses, and evaluating the reliability of product claims. Consumers with stronger GenAI literacy may therefore be more capable of directing the interaction toward relevant attributes and recognizing when an output requires clarification or external verification (Long and Magerko, 2020; Ng et al., 2021).

These capabilities can support consumers’ assessments of the decision process. More focused queries may produce information that is easier to compare, while critical evaluation can help consumers resolve inconsistencies before reaching a choice. Consumers who can use these strategies are likely to feel more confident that they have considered the relevant alternatives and followed a systematic process. GenAI literacy is therefore expected to be positively associated with perceived decision quality.

*H1:* GenAI literacy is positively associated with perceived decision quality.

#### 2.2.2 The mediating role of perceived information overload

GenAI literacy may also shape perceived decision quality through the cognitive demands experienced during information processing. Consumers with stronger literacy can formulate narrower requests, organize generated content around decision criteria, and identify information that can be disregarded. These strategies can reduce unnecessary processing and help consumers maintain a manageable flow of information. Consumers with less developed literacy may find it more difficult to determine which parts of a response are relevant or credible, increasing the effort required to compare alternatives.

Perceived information overload can subsequently influence how consumers evaluate the decision process. A consumer who feels overwhelmed may devote less attention to individual attributes, reduce the number of alternatives considered, or rely on simplifying rules. These responses can leave the consumer less confident that the final choice reflects a systematic assessment of the available information. Lower perceived information overload should allow more cognitive resources to remain available for comparison and evaluation, supporting higher perceived decision quality.

The two relationships form the proposed indirect pathway. Stronger GenAI literacy is expected to be associated with lower perceived information overload, which is in turn expected to be associated with higher perceived decision quality. The mediation hypothesis concerns this pattern of associations within the proposed model.

*H2:* Perceived information overload mediates the association between GenAI literacy and perceived decision quality.

#### 2.2.3 The moderating role of consumer involvement

Consumer involvement refers to the personal relevance or importance that an individual attaches to a product or purchase decision (Zaichkowsky, 1985). Greater involvement generally increases consumers’ willingness to search for information and examine product attributes carefully. In GenAI-assisted shopping, this motivation can lead consumers to ask more detailed questions, compare additional alternatives, and inspect generated recommendations more closely.

The consequences of this additional effort may depend on the consumer’s GenAI literacy. Consumers with stronger literacy can use their knowledge to structure the interaction, focus on relevant information, and evaluate uncertain claims. Under higher involvement, they have greater motivation to apply these skills throughout the decision process. Consumers with lower literacy may expand their information search without having equally effective strategies for organizing and evaluating the resulting content. The difference in perceived information overload between consumers with higher and lower literacy is therefore expected to become more pronounced as involvement increases.

This moderation occurs on the first stage of the proposed indirect pathway. At higher levels of consumer involvement, the negative association between GenAI literacy and perceived information overload should be stronger, producing a larger indirect association between GenAI literacy and perceived decision quality through perceived information overload.

*H3:* Consumer involvement moderates the indirect association between GenAI literacy and perceived decision quality through perceived information overload. Specifically, the negative association between GenAI literacy and perceived information overload is stronger at higher levels of consumer involvement.

## 3 Research design and method

### 3.1 Research design

This study adopted a cross-sectional quantitative survey design to examine the associations among GenAI literacy, perceived information overload, consumer involvement, and perceived decision quality in online shopping. The research model proposes that GenAI literacy is associated with perceived decision quality both directly and indirectly through perceived information overload, while consumer involvement conditions the association between GenAI literacy and perceived information overload. As all focal variables were measured at a single point in time, the empirical analysis evaluates associations consistent with the proposed theoretical mechanism without establishing their temporal or causal order.

Before completing the measures, participants received a contextualizing instruction asking them to consider their general experiences and capabilities when using GenAI tools such as ChatGPT and DeepSeek and to apply these experiences to online shopping activities, including searching for product information, comparing product features, and reading recommendations. Previous use of GenAI in an actual purchase decision was not imposed as a separate eligibility requirement. The responses therefore capture participants’ assessments within a specified GenAI-assisted shopping context.

### 3.2 Sampling and data collection

The sampling frame consisted of college students enrolled at educational institutions in China. University students were selected because they frequently encounter emerging digital services in their studies and everyday activities and are therefore an appropriate population for examining the role of GenAI-related capabilities in online consumer decision-making. Data were collected in January 2026 through Wenjuanxing, a widely used online survey platform in China. Participants were recruited through social media and email invitations using a non-probability convenience sampling strategy. Participation was voluntary, and no financial or material compensation was provided. Before beginning the questionnaire, participants were informed of the purpose of the study and assured that their responses would remain anonymous and confidential.

The final sample comprised 401 respondents. Of these participants, 184 were male (45.89%) and 217 were female (54.11%). Freshmen accounted for 18.70% of the sample, sophomores for 25.94%, juniors for 28.18%, seniors for 18.95%, and graduate students or above for 8.23%. The dataset contained complete responses for all variables included in the analysis, and no cases were removed from the final analytical sample.

### 3.3 Measurement and scaling

The measurement items were adapted from established scales and contextualized for GenAI-assisted online shopping. Unless otherwise indicated, the score for each construct was calculated as the arithmetic mean of its constituent items. This procedure retained the original response metric and assigned equal weight to each item. Higher composite scores represented higher levels of the corresponding construct.

#### 3.3.1 GenAI literacy

GenAI literacy was measured using the ten-item Generative Artificial Intelligence Literacy Scale developed by Gökçearslan et al. (2024). The scale comprises four dimensions: Awareness, Usage, Evaluation, and Ethics. Representative items include “I can identify the technology used in generative AI applications and products,” “I can skillfully use generative AI applications or products to help me in my daily work,” and “I can evaluate the capabilities and limitations of a generative AI application or product after using it for a while.” Responses were recorded on a five-point Likert scale ranging from 1 (strongly disagree) to 5 (strongly agree). The ten items were averaged to form the GenAI literacy score, with higher scores indicating greater GenAI literacy. Cronbach’s α for the scale was 0.940.

#### 3.3.2 Perceived information overload

Perceived information overload was measured using fourteen items adapted from the Shoppers’ Susceptibility to Information Overload Scale developed by Hunter et al. (2024). The questionnaire retained four dimensions of the original instrument: sensitivity to the amount of information, anticipation of negative affect, anticipation of mistakes, and perceived information-processing ability. The original dimension concerning perceived availability of time was not included. Item wording was adapted to situate the informational demands within GenAI-assisted shopping. Representative items include “When using GenAI to assist shopping, there are too many options for me to make a purchase decision” and “When confronted with too much information provided by GenAI, I often select the wrong product”.

Participants responded on a seven-point Likert scale ranging from 1 (strongly disagree) to 7 (strongly agree). The four positively worded information-processing ability items were coded in the reverse direction during questionnaire scoring so that higher values consistently represented greater susceptibility to information overload. The fourteen coded items were then averaged to produce the perceived information overload score. Cronbach’s α for the scale was 0.956.

#### 3.3.3 Perceived decision quality

Perceived decision quality was assessed using four items adapted from Chae and Lee (2013). The items capture respondents’ evaluations of the quality and systematic nature of the decision process in a GenAI-assisted online shopping setting. Examples include “The purchase decision-making process in this online shop with GenAI assistance is systematic” and “This online shop, with GenAI assistance, helps me make highly systematic decisions.” Responses were recorded on a seven-point Likert scale ranging from 1 (strongly disagree) to 7 (strongly agree). The four items were averaged, with higher scores indicating a more favorable evaluation of the decision process. Cronbach’s α for the scale was 0.862.

#### 3.3.4 Consumer involvement and control variables

Consumer involvement was measured using seven items adapted from the Personal Involvement Inventory developed by Zaichkowsky (1985). The instrument uses bipolar semantic-differential items to capture the personal relevance and importance attached to a product or purchase decision. The seven items were scored on a seven-point scale and averaged to form a continuous involvement measure. Higher scores indicated greater consumer involvement. Consumer involvement was retained as a continuous variable in all analyses and was not divided into high- and low-involvement groups. The scale demonstrated satisfactory internal consistency, with a Cronbach’s α of 0.910.

Gender, grade level, and socioeconomic status were included as control variables. Gender was coded as 1 for male and 2 for female. Grade level was coded from 1 to 5, corresponding to freshman, sophomore, junior, senior, and graduate student or above. Socioeconomic status was self-reported on a five-point scale ranging from 1 (extremely low) to 5 (extremely high). The categorical control variables were represented by indicator variables in the multivariate analyses.

### 3.4 Analytical procedure

The analysis was conducted in Stata and proceeded in two stages. The measurement analysis first examined the internal structure of the scales. Exploratory factor analysis using principal-axis factoring and oblique rotation was employed to inspect the pattern of item loadings and identify material cross-loadings. Confirmatory factor analysis was then used to evaluate the hypothesized measurement structure and to compare alternative specifications, including a single-factor model, a correlated construct model, and a multidimensional model representing the first-order dimensions of GenAI literacy and perceived information overload. The multidimensional analysis was used to assess whether the respective dimensions could be represented by aggregate GenAI literacy and perceived information overload scores.

The Likert-scale indicators were treated as approximately continuous and estimated using maximum likelihood. Measurement-model fit was evaluated using the chi-square statistic, comparative fit index (CFI), Tucker–Lewis index (TLI), root mean square error of approximation (RMSEA), and standardized root mean square residual (SRMR). Convergent validity was assessed from the standardized factor loadings, composite reliability (CR), and average variance extracted (AVE). CR was calculated as (Σλ)^2^/[(Σλ)^2^ + Σθ], and AVE as Σλ^2^/[Σλ^2^ + Σθ], where λ denotes a standardized factor loading and θ its corresponding error variance. Discriminant validity was evaluated using the Fornell–Larcker criterion and heterotrait–monotrait ratios. Because the variables were collected from the same questionnaire, Harman’s single-factor analysis and a single-factor CFA model were also examined as diagnostic assessments of common method variance.

The hypotheses were tested using standardized mean composite scores. H1 was evaluated by estimating the association between GenAI literacy and perceived decision quality after controlling for gender, grade level, and socioeconomic status. H2 was tested by estimating the product of the GenAI literacy–information overload path and the information overload–perceived decision quality path. H3 was specified as a first-stage moderated mediation model: the product term between standardized GenAI literacy and consumer involvement was included in the perceived information overload equation, while the perceived decision quality equation included GenAI literacy and perceived information overload. The interaction term was not included as a separate predictor of perceived decision quality because the hypothesis concerns moderation of the first stage of the indirect pathway. Percentile confidence intervals for the indirect effect and the index of moderated mediation were obtained from 3,000 bootstrap samples using a fixed random seed. Statistical significance was evaluated using two-tailed tests with an α level of 0.05.

## 4 Empirical analysis and results

### 4.1 Measurement model assessment

#### 4.1.1 Exploratory and confirmatory factor analyses

An exploratory factor analysis was first conducted on the 35 measurement items using principal-axis factoring with oblique Promax rotation. The Kaiser–Meyer–Olkin measure was 0.962, and Bartlett’s test of sphericity was statistically significant, χ^2^(595) = 9,524.43, *p* < 0.001, indicating that the correlation matrix was suitable for factor analysis. Four factors had eigenvalues greater than 1 and corresponded to GenAI literacy, perceived information overload, perceived decision quality, and consumer involvement. The items loaded between 0.712 and 0.803 on their expected factors. No secondary loading reached 0.300, providing initial support for the proposed four-construct structure.

Confirmatory factor analysis was subsequently performed using maximum-likelihood estimation. The correlated four-factor model provided a good fit to the data, χ^2^(554) = 627.41, *p* = 0.016, RMSEA = 0.018, 90% CI [0.008, 0.025], CFI = 0.992, TLI = 0.991, and SRMR = 0.029. By comparison, a model in which all 35 items loaded on a single factor exhibited poor fit, χ^2^(560) = 4,469.65, RMSEA = 0.132, CFI = 0.577, TLI = 0.550, and SRMR = 0.148. The marked difference between the two models supports the empirical distinction among the four constructs.

Additional analyses examined whether the dimensions of GenAI literacy and perceived information overload could be distinguished empirically. Although the correlated first-order models produced favorable global fit indices, several estimated correlations among the dimensions approached or slightly exceeded 1.00. These boundary estimates indicated that the dimensions did not exhibit sufficient empirical separation in the present sample. The more parsimonious single-factor models produced admissible solutions for both scales, with CFI and TLI values above 0.990 and RMSEA and SRMR values below 0.030. They also yielded lower Akaike and Bayesian information criteria than the corresponding multidimensional models. The subsequent analyses therefore used overall mean scores for GenAI literacy and perceived information overload. Their respective dimensions remain part of the conceptual coverage of each scale but were not treated as distinct variables in the structural model.

#### 4.1.2 Reliability and validity test

As reported in Table 1, all standardized factor loadings were statistically significant and ranged from 0.751 to 0.796 in the confirmatory measurement model. Cronbach’s α ranged from 0.862 to 0.956, composite reliability ranged from 0.863 to 0.956, and average variance extracted values ranged from 0.593 to 0.611. These results exceed the conventional thresholds of 0.700 for internal consistency and 0.500 for convergent validity.

**Table 1:** Standardized factor loadings, reliability, and convergent validity.

Construct	Item	Standardized loading	CR	AVE	Cronbach’s α
GenAI literacy	GenAI1	0.787	0.940	0.611	0.940
	GenAI2	0.780			
	GenAI3	0.784			
	GenAI4	0.792			
	GenAI5	0.763			
	GenAI6	0.796			
	GenAI7	0.777			
	GenAI8	0.791			
	GenAI9	0.784			
	GenAI10	0.760			
Perceived information overload	PIO1	0.771	0.956	0.606	0.956
	PIO2	0.783			
	PIO3	0.783			
	PIO4	0.780			
	PIO5	0.793			
	PIO6	0.772			
	PIO7	0.778			
	PIO8	0.785			
	PIO9	0.780			
	PIO10	0.789			
	PIO11	0.761			
	PIO12	0.751			
	PIO13	0.773			
	PIO14	0.795			
Perceived decision quality	PDQ1	0.780	0.863	0.611	0.862
	PDQ2	0.790			
	PDQ3	0.774			
	PDQ4	0.781			
Consumer involvement	CI1	0.757	0.911	0.593	0.910
	CI2	0.756			
	CI3	0.775			
	CI4	0.782			
	CI5	0.792			
	CI6	0.764			
	CI7	0.761			

CR = composite reliability; AVE = average variance extracted. All standardized factor loadings were statistically significant at *p* < 0.001.

Discriminant validity was evaluated using the Fornell–Larcker criterion and heterotrait–monotrait ratios. As shown in Table 2, the square root of AVE for each construct exceeded its correlations with the other constructs. The HTMT values ranged from 0.372 to 0.523, remaining below the conservative threshold of 0.850. The measurement results therefore support the reliability, convergent validity, and discriminant validity of the four construct scores used in the subsequent analyses.

**Table 2:** Descriptive statistics, correlations, and HTMT ratios.

Variable	M (SD)	1	2	3	4
GenAI literacy	3.31 (1.14)	0.782	0.440	0.452	0.374
Perceived information overload	3.61 (1.32)	−0.417***	0.778	0.523	0.385
Perceived decision quality	4.43 (1.36)	0.406***	−0.475***	0.782	0.372
Consumer Involvement	4.44 (1.29)	0.346***	−0.359***	0.329***	0.770

*N* = 401. Values below the diagonal are Pearson correlations; values above the diagonal are heterotrait–monotrait ratios; diagonal values are the square roots of average variance extracted. ***p < 0.001, **p < 0.01, *p < 0.05.

### 4.2 Common method bias test

Because all focal variables were obtained from the same respondents at a single point in time, common method variance was assessed using two diagnostic procedures. In Harman’s single-factor analysis, the first unrotated factor accounted for 37.69% of the total variance. The proportion was below the commonly used 40% benchmark. The single-factor CFA model also exhibited substantially poorer fit than the correlated four-factor model.

These results indicate that the covariance among the measures cannot be adequately represented by a single common factor. Nevertheless, the statistical diagnostics do not exclude all possible sources of common method variance. The reliance on a single self-reported questionnaire is therefore considered when interpreting the results and is addressed further in the limitations section.

### 4.3 Descriptive statistics and correlation

Table 2 presents the descriptive statistics, bivariate correlations, and heterotrait–monotrait ratios for the four focal constructs. GenAI literacy was positively correlated with perceived decision quality (r = 0.406, *p* < 0.001) and negatively correlated with perceived information overload (r = −0.417, *p* < 0.001). Perceived information overload was negatively correlated with perceived decision quality (r = −0.475, *p* < 0.001). Consumer involvement was positively correlated with GenAI literacy (r = 0.346, *p* < 0.001) and perceived decision quality (r = 0.329, *p* < 0.001), and negatively correlated with perceived information overload (r = −0.359, *p* < 0.001). These bivariate patterns are consistent with the proposed relationships and provide a basis for the subsequent covariate-adjusted analyses.

### 4.4 Hypothesis test

The hypotheses were evaluated using covariate-adjusted path models based on standardized composite scores. Gender, grade level, and socioeconomic status were included as covariates in each equation. The first-stage interaction between GenAI literacy and consumer involvement was specified in the perceived information overload equation, whereas the perceived decision quality equation included GenAI literacy and perceived information overload. Indirect and conditional indirect associations were evaluated using percentile confidence intervals based on 3,000 bootstrap resamples. Given the cross-sectional design, the estimated coefficients are interpreted as conditional statistical associations.

#### 4.4.1 GenAI literacy and perceived decision quality

H1 proposed that GenAI literacy would be positively associated with perceived decision quality. In the covariate-adjusted model, GenAI literacy exhibited a significant positive association with perceived decision quality (β = 0.403, bootstrap SE = 0.043, *p* < 0.001, 95% bootstrap CI [0.322, 0.488]). The model accounted for 17.7% of the variance in perceived decision quality. These results support H1.

#### 4.4.2 Indirect association through perceived information overload

GenAI literacy was negatively associated with perceived information overload (β = −0.418, SE = 0.046, *p* < 0.001). In the perceived decision quality equation, perceived information overload remained negatively associated with perceived decision quality after GenAI literacy and the covariates were taken into account (β = −0.372, SE = 0.047, *p* < 0.001). The corresponding model explained 29.0% of the variance in perceived decision quality.

As shown in Table 3, the indirect association between GenAI literacy and perceived decision quality through perceived information overload was positive and statistically different from zero (β = 0.156, bootstrap SE = 0.026, 95% bootstrap CI [0.108, 0.210]). The direct association between GenAI literacy and perceived decision quality also remained significant (β = 0.248, bootstrap SE = 0.047, 95% bootstrap CI [0.156, 0.342]). This pattern is consistent with partial statistical mediation and supports H2.

**Table 3:** Total, direct, and indirect associations.

Association	β	SE	*p*	95% confidence interval	
				Lower	Upper
Total association	0.403	0.043	<0.001	0.322	0.488
Direct association	0.248	0.047	<0.001	0.156	0.342
Indirect association through perceived information overload	0.156	0.026	<0.001	0.108	0.210

Standardized estimates are reported. Bootstrap standard errors and percentile confidence intervals were based on 3,000 resamples. Gender, grade level, and socioeconomic status were included as covariates.

#### 4.4.3 Moderating role of consumer involvement and conditional indirect association

H3 proposed that the association between GenAI literacy and perceived information overload would vary with consumer involvement. As shown in Table 4, the interaction between GenAI literacy and consumer involvement was negative and statistically significant (β = −0.147, bootstrap SE = 0.047, *p* = 0.002, 95% bootstrap CI [−0.241, −0.059]). The model containing the interaction explained 25.5% of the variance in perceived information overload.

**Table 4:** First-stage moderation and conditional indirect associations.

Analysis and term	Estimate	SE	*p*	95% confidence interval	
				Lower	Upper
Panel A. First-stage moderation model					
GenAI literacy	−0.333	0.047	<0.001	−0.425	−0.240
Consumer involvement	−0.212	0.048	<0.001	−0.306	−0.118
GenAI literacy × consumer involvement	−0.147	0.047	0.002	−0.241	−0.059
Panel B. Simple slopes of GenAI literacy					
Low consumer involvement (−1 SD)	−0.186	0.067	0.006	−0.318	−0.054
High consumer involvement (+1 SD)	−0.480	0.065	<0.001	−0.608	−0.352
Panel C. Conditional indirect associations					
Low consumer involvement (−1 SD)	0.069	0.028	0.012	0.017	0.125
High consumer involvement (+1 SD)	0.179	0.034	<0.001	0.117	0.250
Index of moderated mediation	0.055	0.019	0.005	0.020	0.095

*N* = 401. The dependent variable in Panel A and Panel B is perceived information overload. The first-stage model explained 25.5% of the variance in perceived information overload. Low and high consumer involvement represent one standard deviation below and above the mean, respectively. Standard errors and confidence intervals for the interaction term, conditional indirect associations, and index of moderated mediation were based on 3,000 bootstrap resamples; the remaining standard errors and confidence intervals are model-based. All models included gender, grade level, and socioeconomic status as covariates.

Simple-slope estimates further clarified the interaction. At one standard deviation below the mean of consumer involvement, GenAI literacy was negatively associated with perceived information overload (β = −0.186, SE = 0.067, *p* = 0.006, 95% CI [−0.318, −0.054]). At one standard deviation above the mean, the negative association was stronger (β = −0.480, SE = 0.065, *p* < 0.001, 95% CI [−0.608, −0.352]). Thus, the negative association between GenAI literacy and perceived information overload became stronger as consumer involvement increased. The interaction pattern is illustrated in Figure 2.

**Figure 2:**
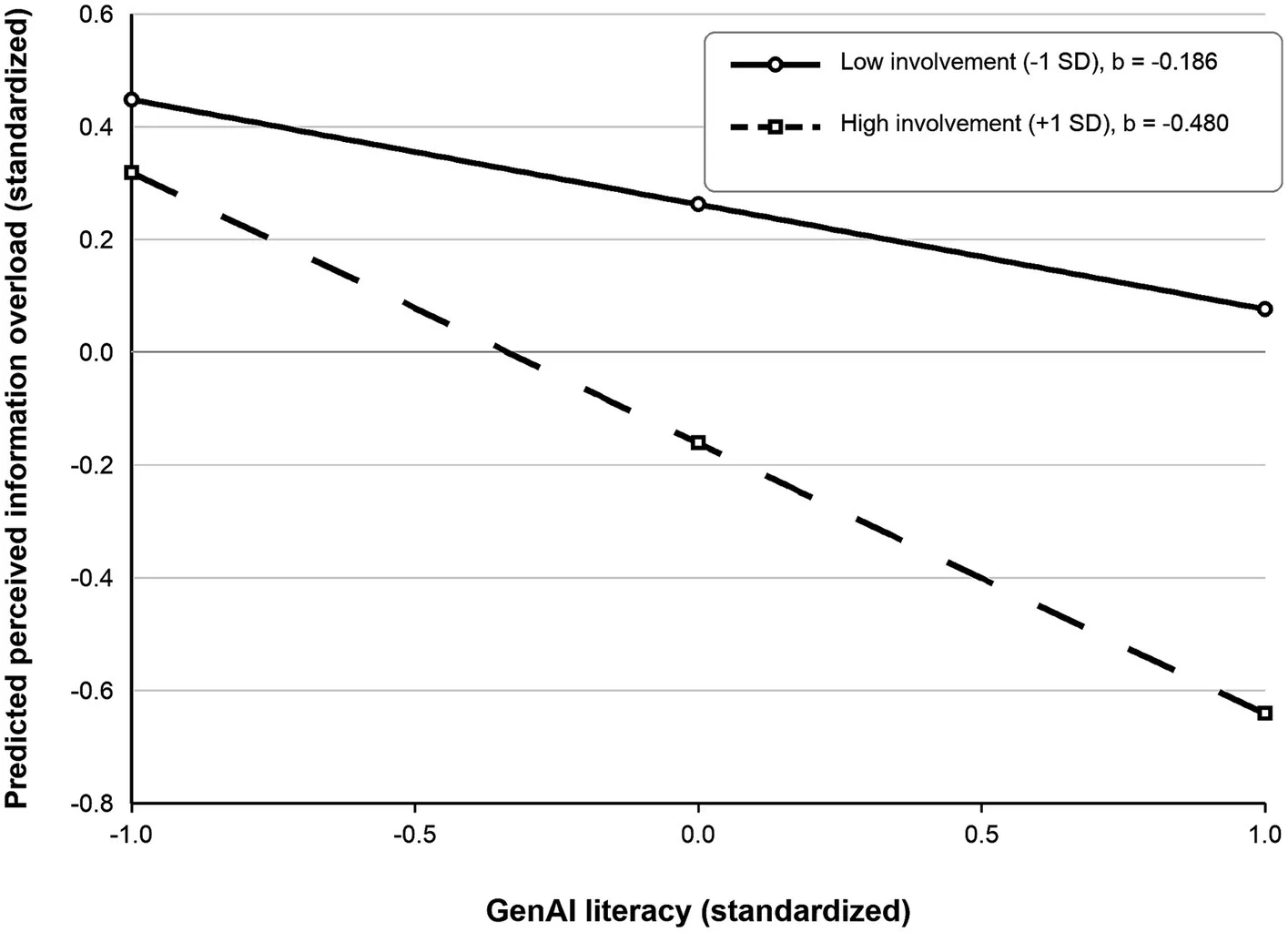
Moderating effect of consumer involvement on the association between GenAI literacy and perceived information overload. Predicted values were calculated from the covariate-adjusted first-stage moderation model. Low and high consumer involvement represent one standard deviation below and above the mean, respectively.

The index of moderated mediation was positive, and its bootstrap confidence interval excluded zero (index = 0.055, bootstrap SE = 0.019, 95% bootstrap CI [0.020, 0.095]). The conditional indirect association between GenAI literacy and perceived decision quality through perceived information overload was 0.069 at low consumer involvement (bootstrap SE = 0.028, 95% bootstrap CI [0.017, 0.125]) and 0.179 at high consumer involvement (bootstrap SE = 0.034, 95% bootstrap CI [0.117, 0.250]). The indirect association was therefore stronger at higher levels of consumer involvement, supporting H3.

## 5 Discussion

### 5.1 Discussion of findings

This study examined the associations among GenAI literacy, perceived information overload, and perceived decision quality in GenAI-assisted shopping, together with the moderating role of consumer involvement. The findings offer several insights into how consumers’ capabilities and motivational context are related to their experiences of GenAI-supported decision making.

The results show a significant positive association between GenAI literacy and perceived decision quality. In the generative AI context, access to advanced tools alone does not necessarily translate into more favorable evaluations of a decision. Consumers with higher GenAI literacy may be better able to recognize limitations in generated content, identify inconsistencies, and compare information relevant to a purchase. These capabilities may help them organize GenAI outputs into more useful decision inputs and increase their confidence in the decision process. The finding extends research on algorithmic recommendations by drawing attention to the role of consumers’ own capabilities in shaping their evaluations of GenAI-assisted decisions.

Perceived information overload was negatively associated with perceived decision quality and accounted for a significant indirect association between GenAI literacy and perceived decision quality. In conventional online shopping, information overload is often associated with the volume and complexity of available information. GenAI-assisted shopping may introduce additional processing demands because generated content can appear plausible while containing uncertain or inconsistent information that requires verification. Although such demands have been described as verification fatigue, they were captured in this study as part of the broader experience of perceived information overload; whether they constitute a distinct psychological construct remains an open empirical question. The observed pattern is consistent with Li et al. (2025), who linked information overload in GenAI settings to heuristic reliance, and with broader concerns about the limitations of generated content discussed by Dwivedi et al. (2023). From an information-processing perspective, GenAI literacy may function as a consumer capability associated with more manageable information-processing demands and, in turn, more favorable perceptions of decision quality.

The moderation analysis further showed that the negative association between GenAI literacy and perceived information overload was present at both lower and higher levels of consumer involvement, but was stronger when involvement was higher. This pattern is consistent with prior research that considers both ability and motivation in AI use (Liu et al., 2025; Sassenberg et al., 2026). When a purchase decision is more personally relevant, consumers may be more motivated to apply their GenAI-related knowledge when scrutinizing generated information and managing its processing demands. At lower levels of involvement, the motivation to apply the same capabilities may be weaker. The finding is also consistent with Gong’s (2026) observation that knowledge of intelligent technologies is associated with lower cognitive strain, while indicating that this association may vary with the motivational relevance of the decision.

### 5.2 Theoretical implications

This research contributes to the literature on information systems and consumer behavior in two main ways.

First, this study extends the application of Information Processing Theory to GenAI-assisted consumer decision making. Previous applications of IPT in consumer research have largely emphasized processing demands arising from information volume and information redundancy (Li, 2014). The present findings suggest that, in GenAI-assisted shopping, perceived processing demands may also be associated with the need to evaluate plausible but potentially uncertain generated content. Verification demands are understood here as one feature of the GenAI information environment that may contribute to perceived information overload. Whether these demands constitute a distinct psychological construct remains a question for future research. By showing that GenAI literacy is negatively associated with perceived information overload and that perceived information overload is negatively associated with perceived decision quality, the study draws attention to the role of consumers’ capabilities alongside the characteristics of the information environment.

Second, this study extends research on GenAI literacy by examining its relevance to consumer decision processes. Previous research has primarily conceptualized AI literacy as a multidimensional competence for understanding and interacting with AI systems (Long and Magerko, 2020; Ng et al., 2021). The present findings indicate that consumers’ overall GenAI literacy is associated with lower perceived information overload and higher perceived decision quality. GenAI literacy may therefore serve as a cognitive resource that helps consumers engage with generated content and manage the processing demands they experience, particularly when the decision is personally relevant. This perspective complements research focused on AI adoption and operational competence by highlighting how consumers’ capabilities are related to their subjective evaluations of GenAI-assisted decision making.

### 5.3 Practical and policy implications

The findings suggest several practical and policy considerations for e-commerce platforms, AI developers, educational institutions, and consumer-protection bodies.

First, platforms and AI developers could adapt the presentation of GenAI-generated information to the level of consumer involvement. The negative association between GenAI literacy and perceived information overload was present at both lower and higher levels of involvement, but was stronger when involvement was higher. For more personally consequential purchases, AI assistants could support evaluation by providing traceable sources, structured comparisons, and concise indications of uncertainty. For routine purchases, shorter default responses with optional access to further detail may help limit unnecessary processing demands. Such design features would allow consumers to obtain different levels of information without assuming that the same response format is appropriate for every decision.

Second, the findings also have implications for consumer education and digital-literacy policy. Training initiatives could extend beyond basic operational skills, such as prompt formulation, to include source evaluation, recognition of uncertainty, comparison of alternative outputs, and awareness of potential bias. Educational institutions and consumer-protection bodies could incorporate these competencies into guidance on responsible GenAI use. Because the present study examined perceived rather than objective decision quality, the effectiveness of such initiatives should also be evaluated against behavioral measures of decision performance.

### 5.4 Limitations and future research

Several limitations should be considered when interpreting the findings and provide directions for future research.

To begin with, the sample consisted exclusively of university students in China recruited through convenience sampling. This relatively homogeneous population limits the extent to which the findings can be generalized to consumers of different ages, educational backgrounds, socioeconomic positions, or cultural settings. Moreover, previous use of GenAI in an actual purchase decision was not imposed as an eligibility requirement. Participants were asked to consider their general experience with GenAI within specified shopping activities, so the findings may not fully represent consumers with established experience using GenAI for product search, comparison, recommendation, or purchase decisions. Future studies could establish clearer experience-based inclusion criteria and replicate the model in broader consumer samples and cultural contexts.

Second, all focal variables were measured through a self-reported questionnaire administered at a single point in time. Although the diagnostic analyses indicate that a single common factor does not adequately account for the covariance among the measures, these procedures cannot exclude all possible sources of common method variance. The cross-sectional design also prevents conclusions about temporal or causal ordering. The estimated indirect and conditional indirect associations should therefore be interpreted as statistical relationships consistent with the proposed theoretical mechanism. Longitudinal, experimental, and multi-source designs would provide stronger evidence regarding the direction and development of these relationships.

Finally, the outcome measure captures perceived decision quality rather than objective decision accuracy or performance. Perceived information overload was likewise assessed through self-report, while verification-related demands were considered part of this broader construct rather than independently measured and validated. Future research could combine self-reported perceptions with behavioral choice tasks, accuracy measures, response times, and process-tracing methods such as eye tracking. Separate conceptual development and psychometric validation would also be required before verification fatigue could be treated as a distinct psychological construct.

## 6 Conclusion

Generative AI is changing how consumers search for, compare, and evaluate product information. Drawing on Information Processing Theory, this study examined the associations among GenAI literacy, perceived information overload, and perceived decision quality, together with the moderating role of consumer involvement. GenAI literacy was positively associated with perceived decision quality and negatively associated with perceived information overload. Perceived information overload accounted for a significant indirect association between GenAI literacy and perceived decision quality, while the negative association between GenAI literacy and perceived information overload became stronger as consumer involvement increased.

Taken together, the study shifts attention from consumers’ access to GenAI tools toward their capacity to understand and critically evaluate generated information and the decision conditions under which that capacity is applied. The findings suggest that transparent information presentation and critical GenAI literacy are relevant to how consumers experience and manage information-processing demands in AI-assisted shopping, particularly when purchase decisions are personally relevant.

## Data Availability

The raw data supporting the conclusions of this article will be made available by the authors, without undue reservation.
